# Ferromagnetic Mass Localization in Check Point Configuration Using a Levenberg Marquardt Algorithm

**DOI:** 10.3390/s91108852

**Published:** 2009-11-04

**Authors:** Roger Alimi, Nir Geron, Eyal Weiss, Tsuriel Ram-Cohen

**Affiliations:** Propulsion Physics Division, Soreq NRC, Yavne 81800, Israel; E-Mails: roger@soreq.gov.il (R.A.); nirg@soreq.gov.il (N.G.); tsuriel@soreq.gov.il (T.R.C.)

**Keywords:** magnetic moment localization, magnetic sensors, Levenberg Marquardt Algorithm

## Abstract

A detection and tracking algorithm for ferromagnetic objects based on a two stage Levenberg Marquardt Algorithm (LMA) is presented. The procedure is applied to localization and magnetic moment estimation of ferromagnetic objects moving in the vicinity of an array of two to four 3-axis magnetometers arranged as a check point configuration. The algorithms first stage provides an estimation of the target trajectory and moment that are further refined using a second iteration where only the position vector is taken as unknown. The whole procedure is fast enough to provide satisfactory results within a few seconds after the target has been detected. Tests were conducted in Soreq NRC assessing various check point scenarios and targets. The results obtained from this experiment show good localization performance and good convivial with “noisy” environment. Small targets can be localized with good accuracy using either a vertical “doorway” two to four sensors configuration or ground level two to four sensors configuration. The calculated trajectory was not affected by nearby magnetic interference such as moving vehicles or a combat soldier inspecting the gateway.

## 1. Introduction

An array of magnetic flux sensors is often used to monitor changes in earth's magnetic field. Algorithms for tracking ferromagnetic targets in a sensors array may generally be classified into three categories: the direct approach, in which the important work of Wynn's [1] deserves special attention as well as [2-7], the statistical approach [8-10] and the heuristic approach [11-13].

The direct or deterministic approach utilized analytical solution of the magnetic flux equations for the six unknowns that characterize the target. Three variables characterize the position and another three the magnetic moment. A variety of methods are put to practice, some of which requiring dedicated hardware. These methods can either employ fundamental formula or rely on intelligent numerical schemes. The chief advantages the direct approach methods present are their realization simplicity and the rapid processing time. These advantages commonly prevail when real time analysis is required at the cost of limited localization accuracy. These methods are also limited to small scale systems as they generally are sensitive to “noisy” environment.

On the other hand, statistical and heuristic approaches generally provide more accurate solutions at the cost of extensive computational resources. As a result, statistical and heuristic approaches are often used as post processing algorithms, yielding high precision at the cost of prolonged response time.

This paper presents the performance of a particularly designed localization algorithm for a check point configuration. This application requires precise (on-body) localization of ferromagnetic mass moving in a controlled passageway. The sensors can either be located on ground level or on a vertical mount (sand bags, doorways etc.).

As implied by the application, response time is of great importance while on body localization precision requirements are less stringent. Target location includes height and body flank, left or right, at which the object is carried. As deterministic method is more suitable for this application, we decided to implement a non linear least square searching algorithm to get the first localization estimation. Applying simple physical considerations to quickly refine the first estimation provides improved precision.

The paper is outlined as follows: after a brief recall of the magnetic anomaly characterization theory, we describe the two stages localization algorithm. The experimental setup is then presented together with a detailed event analysis. Finally, a summary of the results for various configurations of the check point geometry is given.

## 2. The Localization Procedure

### 2.1. The Levenberg Marquardt Algorithm

A detection trigger is given by a detection algorithm [14-16]. That triggering algorithm is based on the detection of abnormal distortions of earth's magnetic field due to motion of ferromagnetic mass. After detection the localization algorithm is initiated.

**Figure 1.**
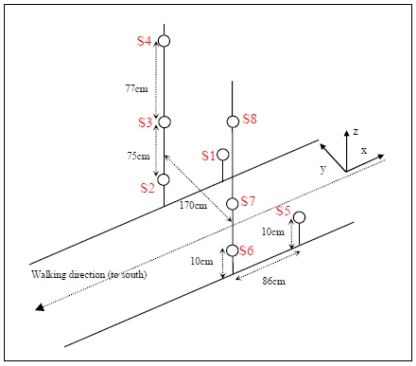
Schematic view of the check point. The relative distance between the sensors are indicated.

A magnetic field *B⃗* created by a ferromagnetic target with a moment *m⃗* at a distance *r⃗* is given by the relation (1):

(1) $$\vec{B}(\vec{m},\vec{r})=\frac{3\mu_{0}}{4\pi }\left[\frac{(\vec{m}\cdot \vec{r})\vec{r}}{R^{5}}-\frac{\vec{m}}{R^{3}}\right]$$

where *m⃗* = (*M_x_,M_y_,M_z_*) and R=|*r⃗*|. In our sensors array the distance *r⃗* depends of course on the (fixed) sensors position. Therefore for each sensor *r⃗* = *r⃗_source_* - *r⃗_sensor_*. A unique reference frame is applied for all calculations. It is shown in Figure 1 below. It is commonly accepted as a rule of thumb that if the distance between the source center and the sensor is at least 2.5 times larger than the largest dimension of the source, then the source may be considered as a magnetic dipole. Our tested sources are approximately 10-15cm long. It is easily shown from Figure 1 that our setup indeed fulfils this condition.

Expanding the field including its three components yields (2):

(2) $$\left|\begin{array}{c} B_{x}\\ B_{y}\\ B_{z} \end{array}\right|=\frac{\mu_{0}}{4\pi R^{5}}\left|\begin{array}{ccc} 3x^{2}-R^{2} & 3xy & 3xz\\ 3yx & 3y^{2}-R^{2} & 3yz\\ 3zx & 3zy & 3z^{2}-R^{2} \end{array}\right|\left|\begin{array}{c} M_{x}\\ M_{y}\\ M_{z} \end{array}\right|$$

The Earth magnetic field is filtered out by the sensor itself (analog high pass filter at 0.003 Hz). The problem of localization may be expressed in terms of a classical inverse problem. Given the magnetic field measured by a well positioned sensor at time t one may wish to find the source position and moment that produces the measured field. From a strict mathematical point of view since we have six unknowns (*x,y,z,M_x_,M_y_,M_z_*) we must have—at least—six equations to solve the problem. The equations are provided by the data measured by two three-axial magnetic sensors.

Nevertheless, the high non-linear characteristic of the equations and the presence of noise, induce ambiguities that necessitate processing additional data. These data are obtained by adding sensors to the grid. This leads to a system of equations that become over determined (more equations than unknowns).

It has been shown [17] that least squares method may be efficiently applied to get approximate solutions to an over determined system. In our case the non linearity of the magnetic equations suggests the use of non-linear least squares. This method often utilizes iterative procedures for the functional error minimization. In order for the algorithm to converge efficiently and provide precise solution it must suit the mathematical and physical characters of the problem.

The Levenberg-Marquardt Algorithm (LMA) is an iterative algorithm that solves the non-linear least square problem [18-20]. It appears to be especially well adapted to our physical system [21]. The algorithm interpolates between the Gauss-Newton algorithm (GNA) and the method of steepest descent. The LMA is more robust than the GNA, which means that in many cases it finds a solution even if it starts very far off the final minimum. On the other hand, for “well-behaved” functions and reasonable starting parameters, the LMA tends to be a bit slower than the GNA.

Given a set of empirical data pairs (*t_i_,y_i_*) (where *y_i_* is the measured data point at time *t_i_*), we optimize the parameters **p** of the model curve *f*(*t*|**p**) so that the sum of the squares of the deviations (3) becomes minimal:

(3) $$\text{S}(\text{p})=\sum_{\text{i}=1}^{\text{m}}{\left[{\text{y}}_{\text{i}}-\text{f}({\text{t}}_{\text{i}}|\text{p})\right]}^{2}$$

In our application **p** = (*x,y,z,M_x_,M_y_,M_z_*). In order to start a minimization process, an initial guess of the parameter vector **p** must be provided. In our case we took the first guess equal as the origin of the axis and zero moment.

In every iteration step, the parameter vector **p** is replaced by a new estimate **p** + **q**. To determine **q**, the functions *f_i_(***p** + **q***)* are approximated by their linearization (4):

(4) f(p+q)≈f(p)+J(q)

where **J** is the Jacobian of **f** at **p**.

At a minimum of the sum of squares **S**, we have ∇*_q_S* = 0. Differentiating the square of the right hand side of the equation above and setting to zero leads to (5):

(5) $$({\text{J}}^{\text{T}}\text{J})\text{q}=-{\text{J}}^{\text{T}}\text{f}$$

from which **q** can be obtained by inverting **J^T^J**. This is the Gauss Newton scheme, which is most efficient when we are close to the solution. The key to applying the LMA is to replace this equation with a ‘damped version’ (6):

(6) $$({\text{J}}^{\text{T}}\text{J}+\lambda \text{I})\text{q}=-{\text{J}}^{\text{T}}\text{f}$$

The positive damping factor λ is adjusted each iteration. If reduction of **S** is rapid a smaller value of λ can be used getting the algorithm closer to the GNA, whereas if an iteration gives insufficient reduction in the residual result, λ can be increased leaning closer to the gradient descent direction.

### 2.2. The Tracking Algorithm

After detection trigger has been given by the detection algorithm a relatively short portion of the signal is filtered and cleaned from bias and trends. The next step is to isolate the part of the path that corresponds to the sensor network crossing event contained by the entire data. If a distracting target interferes, it may be then truncated out of the analysis. Only these data are processed for localization. Local false minimum is avoided by applying a simple annealing procedure together with the LMA. This first round calculates the target trajectory for the six component vector (*x,y,z,M_x_,M_y_,M_z_*). At this stage a robust statistic analysis is applied in order to extract an average value of the moment components assuming these values remain constant during the gate crossing. This assumption is adequate for the total magnetic moment analysis but not necessarily for its projected values (*M_x_,M_y_,M_z_*). However, it is fair to assume that during the few seconds the path is analyzed the metallic object does not rotate significantly and all moment components remain constant. A second LMA round follows solving only the three path variables. Figure 1 depicts the trajectory; x coordinate represent path progression, z coordinate gives the height at which the metallic object is carried and the sign of the y coordinate indicate the side on the body where the object is placed. Note that y and z must be found with high precision in order to fit the application requirements.

### 2.3. Experimental Setups

A checkpoint passageway was constructed by stacking sand bags and carefully placing three-axis sensitive magnetic fluxgates sensors (Bartington Mag634). Eight sensors were installed in the checkpoint covering all relevant sensors configurations. See Figure 1 for a schematic. The reference system places the axis origin (0,0,0) on the ground at equal distance between S2 and S6. Directions are shown in the figure.

The check point was superfluously constructed with an excess of magnetometers allowing selection of certain sensors data according to the analyzed check point configuration. Two main configurations were analyzed: Vertical, where the sensors are located on a vertical mount as in a doorway and Horizontal, where they are all positioned on ground level. For each configuration the number of participating sensors can be selected according to the configuration. The sensors location and their symbols used in this work are shown in red.

In the vertical setup sand bags provide a doorway through which the inspected person is walking. The doorway width was selected to be 170 cm. For this configuration six sensors [S2, S3, S4, S6, S7 and S8] are located at positions shown in Figure 1. The localization algorithm can make use of two to six sensors among the six available. The localization performance varies with the number of sensors actually used.

In the horizontal setup the inspected person is passing through the sensors network while all sensors are on ground level. The sensors are placed on the ground creating a passage through which the person under test is walking. For this configuration we consider four sensors, namely S1, S2 S5 and S6 (see Figure 1). In this case localization algorithm can make use of two to four sensors out of the four available.

The experiment procedure is as follows: the inspected person carries a ferromagnetic object at a specific location on his body. The person stands five meters north to the gate. He then starts walking towards the gate at an approximate velocity of 1m/s and crosses the check point. He stops ten meters after the gate. During this time the signals measured by the entire sensor network are recorded and stored on a PC. Although the data can be processed online, at this stage of the algorithm development and optimization it was more convenient to analyze the signals outside the experiment field.

### 2.4. Tracking Demonstration

As a typical example we show the detailed analysis of the localization of a small metallic object carried at the left side breast of the inspected person (about 1.3 m above ground).

**Figure 2.**
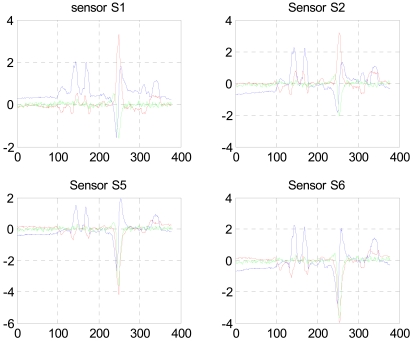
Magentic flux raw data of the crossing of a small metallic object in the left armpit. Units for the x axis are sample points (sp), and the y axis units are nano-Teslas (nT).

Figure 2 shows the raw data measured by sensors S1, S2, S5 and S6. Units for the x axis are sample points (sp), at a sampling rate of 10 Hz, and the y axis units are nano-Teslas (nT). In each plot *B_x_*, *B_y_* and *B_z_* are shown in blue, red and green respectively. During the crossing event a distracting target (a person carrying a large ferromagnetic object simulating an armed guard) is in motion 5 m south of the sensors array. The distracting target signal is clearly visible between 100 and 200 sp in *B_x_*, and *B_y_* in all sensor data.

The first step is to clean the data from high frequency noise (see *B_x_* in sensor S2 for instance) and to isolate the crossing event. This can be done easily by a simple searching procedure since we observe current position of the crossing person.

**Figure 3.**
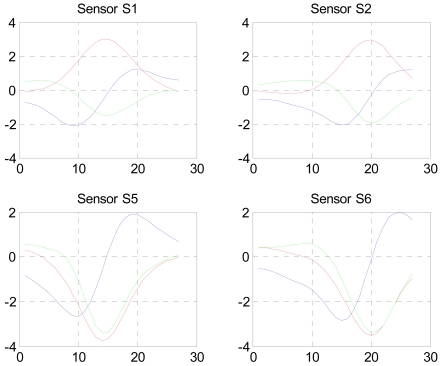
Isolated relevant data shown in Figure 2.

The result is shown in Figure 3 below.

**Figure 4.**
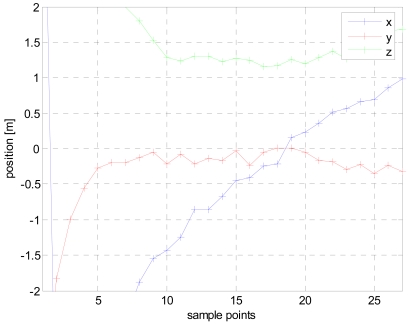
Calculated trajectory after the first round of LMA.

**Figure 5.**
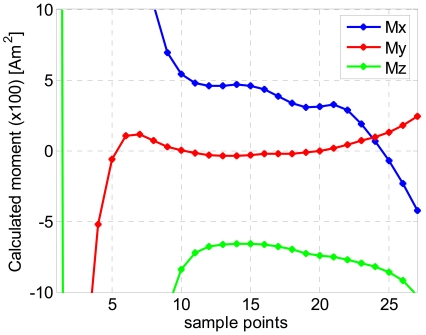
The calculated magnetic moment after the first round of LMA.

Only 26 data points have been selected for the localization procedure and are used by the LMA. Figure 4 shows the results of the first LMA round for the (x,y,z) coordinates and Figure 5 shows the calculated magnetic moments at the same time. The crossing event occurs between 15 and 20 sp and an average value of the moments is extracted within this interval.

**Figure 6.**
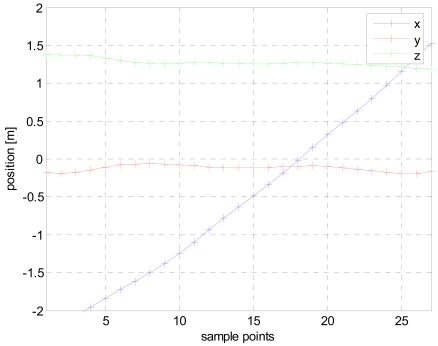
The calculated trajectory after the second round of LMA.

The results of the first iteration are then loaded by the second iteration procedure at which only position vector is being calculated. The modified vector can be seen in Figure 6 depicting straight and stable trajectories for the three coordinates. X axis starts about 2 meters north, cross the gate amid 15 to 20 sp and terminates 1.5 m south. A constant negative offset from the center of the path is clearly visible in the Y coordinate indicating that the ferromagnetic mass is probably located in the left flank of the body. Finally the Z coordinate exhibits an almost constant value of 1.27 m above ground, which correctly corresponds to the breast of the crossing person.

## 3. Algorithm Performance Analysis

In order to test the algorithm performance a series of controlled tests were conducted. Over 100 trajectories were recorded in which several parameters were varied: three different objects (overall mass of 1, 0.7 and 0.5 kg), carried at three different heights, and flank of the person crossing the check point. Targets size may represent real threats expected at check points such as a hand gun, shrapnel pack and a dagger, respectively. In almost all cases controlled distracting targets were moving about during the test. The results clearly show that they had little effect on the performance of the algorithm. For the horizontal configuration, two sub-cases were considered: two sensors (S1 and S5) and four sensors (S1, S2, S5 and S6). For the vertical configuration, two sub-cases were considered: three sensors (S2, S3 and S6) and four sensors (S2, S3, S6 and S8). For each configuration we have calculated the cumulative distribution function (CDF) of the trajectory height (Z coordinate) and flank (Y coordinate) as evaluated by the localization algorithm.

We summarize the results for the height parameter in Table 1. The results in the table present the CDF at a distance of 25 cm deviation from the actual height (Z coordinate) and 5cm deviation for the flank (Y coordinate). A result of 90% indicates that in 90% of the cases the object was localized at a position that falls within 25 cm (for Z and 5 cm for Y) of the true position.

Not surprisingly the vertical configuration yields better results than the horizontal. This is expected as the ferromagnetic object is crossing along the Z axis which is not well sampled by the horizontal configuration.

Nevertheless, using four sensors in the horizontal setup still yields satisfactory predictions. The flank of the object was also evaluated by the localization algorithm. The CDF to find the true side is shown in Table 2 below.

It is interesting to see that for both configurations the less the number of sensors, the better the results. We suggest the following explanation. In the horizontal setup adding two more sensors to the array does not change anything to the symmetry relative to relevant axis (Y axis). However, in the vertical setup three and four sensors configurations have a different symmetry (relative to Y axis) that improves the results, specifically in the three sensors geometry. This hypothesis may be scrutinized by additional testing.

Finally, the magnetic moments provided by the algorithm are shown in Table 3. One may see that the object magnetic moment has been well characterized by the algorithm and can be classified in either large (>5) medium (>2) or small size (<2). Note that for the magnetic moment, both configurations yield similar results and in both setups the more sensors the more accurate the results. Also note that “medium” targets standard deviation is relatively large as may be expected from an assortment of randomly oriented sharpnels.

## 4. Conclusions

A tracking algorithm that localizes ferromagnetic targets is presented. The “engine” of the algorithm is a Levenberg-Marquardt scheme that appears especially well adapted to the physical and mathematical nature of the problem.

A preliminary test of a precise localization of ferromagnetic objects in a check point walkway was performed and analyzed. Although the algorithm can be further improved, the results are already very satisfactory. The tracking procedure is easily implemented and provides the localization parameters and moment estimation of the object within less than 3 seconds after the crossing of the check point.

In most cases (over 90%) both height and flank are located successfully (within 25 cm of the exact position and oin the correct side of the body). The calculated moment of the object is also extracted with good accuracy for target size classification. This work tested only a single ferromagnetic mass carried by the person going through the check point. In future work we plan to address multiple on body targets localization.

## Figures and Tables

**Table 1.** The height localization probability (for a distance of 25 cm from the exact position).

**Target**	**Horizontal setup**		**Vertical setup**	
	**2 sensors**	**4 sensors**	**3 sensors**	**4 sensors**
Large	80%	90%	100%	100%
Medium	60%	85%	90%	100%
Small	45%	75%	100%	100%

**Table 2.** The flank localization probability.

**Target**	**Horizontal setup**		**Vertical setup**	
	**2 sensors**	**4 sensors**	**3 sensors**	**4 sensors**
Large	90%	80%	95%	90%
Medium	85%	80%	100%	100%
Small	70%	65%	95%	80%

**Table 3.** The mean and standard deviation calculated values of the moment for all configurations. Units are 100*A·m^2^.

	**Horizontal setup**		**Vertical setup**	
	**2 sensors**	**4 sensors**	**3 sensors**	**4 sensors**
Large	6.4 ±1.8	6.9 ±1.5	6.3 ±1.3	6.1 ±1.3
Medium	2.6 ±2.0	2.0 ± 1.7	2.7 ±0.9	2.4 ±0.7
Small	1.1 ± 0.8	0. 8 ± 0.4	0.7 ± 0.4	0.7 ± 0.2
